# Comparative de novo transcriptomics and untargeted metabolomic analyses elucidate complicated mechanisms regulating celery (*Apium graveolens* L.) responses to selenium stimuli

**DOI:** 10.1371/journal.pone.0226752

**Published:** 2019-12-30

**Authors:** Chenghao Zhang, Baoyu Xu, Cheng-Ri Zhao, Junwei Sun, Qixian Lai, Chenliang Yu

**Affiliations:** 1 Institute of Agricultural Equipment, Zhejiang Academy of Agricultural Sciences, Hangzhou, China; 2 Department of Horticulture & Landscape Architecture, Agricultural college of Yanbian University, Yanji, China; 3 College of Modern Science and Technology, China Jiliang University, Hangzhou, China; 4 Key Labortatory of Creative Agricultrue, Ministry of Agriculture, Zhejiang Academy of Agricultural Sciences, Hangzhou, China; University of Naples Federico II, ITALY

## Abstract

Presently, concern regarding the effects of selenium (Se) on the environment and organisms worldwide is increasing. Too much Se in the soil is harmful to plants. In this study, Illumina RNA sequencing and the untargeted metabolome of control and Se-treated celery seedlings were analyzed. In total, 297,911,046 clean reads were obtained and assembled into 150,218 transcripts (50,876 unigenes). A total of 36,287 unigenes were annotated using different databases. Additionally, 8,907 differentially expressed genes, including 5,319 up- and 3,588 downregulated genes, were identified between mock and Se-treated plants. “Phenylpropanoid biosynthesis” was the most enriched KEGG pathway. A total of 24 sulfur and selenocompound metabolic unigenes were differentially expressed. Furthermore, 1,774 metabolites and 237 significant differentially accumulated metabolites were identified using the untargeted metabolomic approach. We conducted correlation analyses of enriched KEGG pathways of differentially expressed genes and accumulated metabolites. Our findings suggested that candidate genes and metabolites involved in important biological pathways may regulate Se tolerance in celery. The results increase our understanding of the molecular mechanism responsible for celery’s adaptation to Se stress.

## Introduction

At present, the effects of selenium (Se) on the environment and organisms worldwide are receiving increased attention. Se is an important trace element in many organisms, including humans and animals [1]. The biological functions of Se have become gradually clear to human health, over the past decade [2]. In recent years, applications of Se fertilizers and the development of agricultural in Se-rich areas have attracted interest. However, the difference between a Se deficiency and Se poisoning in the human body is relatively small, and the risk of Se poisoning occurs if the daily intake is more than 400 μg [3]. Se, as a potential toxic substance in higher plants, has been studied in the context of its beneficial effects [4]. At appropriate concentrations, Se promotes plant growth, participates in antioxidative defenses and increases resistance to heavy-metal stress, but at higher concentrations, it causes oxidative damage, in the form of Se-amino acids, which incorporate nonspecifically into proteins [5].

The total amount of Se absorbed and accumulated by plants is correlated with the total amount of Se in the soil [6], but the correlations with the form and valence of Se in the soil are greater [7–9]. Se mainly exists in four stable oxidative valences in soil: selenate (+6), selenite (+4), elemental selenium (0) and selenide (-2) [10,11]. Selenate and selenite selenium are the main forms taken up and utilized by plants [12,13]. Selenate is absorbed by sulfate (S) transporters because Se and S are similar chemically [14,15]. The overexpression of *Arabidopsis thaliana* Sultr1;2, a high-affinity S transporter, can lead to the substantial absorption of selenate[16]. *Sultr2*, encoding a low affinity sulfate transporter, participates in the transfer from roots to shoots, irrespective of the Se supply or an S deficiency [17,18]. However, the mechanism of selenite uptake is not clear. The expression levels of rice (*Oryza sativa*) NIP2;1, a silicon influx transporter, as well as OsPT2 and OsPT8 (two phosphate transporters), are correlated with selenite uptake [19–21].

High Se concentrations can retard plant growth and development, reduce protein synthesis and increase oxidative pressure [22]. It is necessary to adopt new methods to reveal the mechanisms of Se tolerance and accumulation. Omics technology provides new opportunities to understand the organization and function of Se in complex systems [23]. With the rapid development of new-generation sequencing tools, RNA-Seq technology has been widely used and has rapidly become a powerful means of systematically studying transcriptional regulation. *De novo* transcriptome sequencing technology provides a fast and inexpensive method for investigating the genetic information of non-model plant species whose genomic information is not available [24]. Metabolomics approaches screen all the small molecular metabolites of an organism or a cell in a specific physiological period both qualitatively and quantitatively.

Celery (*Apium graveolens* L.) belongs to Apiaceae, which originated in the Mediterranean coast of Europe, Sweden, Egypt and other regions, and is widely cultivated because of its rich nutrient, low caloric, high medicinal characteristics [25,26]. In this study, the combined analyses of the transcriptome and metabolome of control and Se-treated celery seedlings were performed. The findings suggested that candidate genes and metabolites involved in important biological pathways might regulate Se tolerance in celery. The results increase our understanding of the molecular mechanisms of celery’s adaptive responses to Se stress.

## Materials and methods

### Plant materials and treatment

The celery (*A*. *graveolens* L. cv. ‘Hangzhou lvqing’, purchased from Hangzhou fengke seed company) seedlings used in this study were grown in a hydroponic pool of Hoagland’s nutrient solution at the Zhejiang Academy of Agricultural Sciences in Zhejiang Province, China under natural conditions. Six-week-old celery seedlings were used for the treatment. Seedlings were grown in Hoagland’s solution containing 0 (mock) or 100 ppm Na_2_SeO_4_. After 48 h, stems were collected for RNA and metabolites extraction.

### Analysis of hydrogen peroxide (H_2_O_2_), Chlorophyll, malondialdehyde (MDA) and total flavonoids contents

The content of H_2_O_2_ measured using a hydrogen peroxide assay kit (Code: A064-1-1, Nanjing Jiancheng Bioengineering Institute, Nanjing, China). H_2_O_2_ reacts with molybdic acid to form a complex, which was measured at 405 nm. Total chlorophyll was extracted in 95% (v/v) ethanol, and then calculated by determining the absorbance at 470 nm, 649 nm and 665 nm. MDA was determined by MDA assay kit (for plant) (Code: A003-3-1, Nanjing Jiancheng Bioengineering Institute, Nanjing, China), which based on the spectrophotometer measurement of a red-complex produced during the reaction of thiobarbituric acid. The content of total flavonoids was assessed using plant flavonoids test kit (Code:A142-1-1, Nanjing Jiancheng Bioengineering Institute, Nanjing, China). In alkaline nitrite solution, flavonoids and aluminium ions form a red complex with characteristic absorption peaks at 502 nm, which can be used to calculate the content of flavonoids in samples. All of the procedures were carried out following the manufacturer’s protocols.

### RNA isolation, library construction and sequencing

The total RNAs from the six samples were extracted using RNAiso plus (9108, Takara, Dalian, China) according to the manufacturer’s protocol and treated with DNase I (2270, Takara). RNA degradation and contamination were monitored on 1% agarose gels. RNA purity was determined using the NanoPhotometer^®^ spectrophotometer (Implen, CA, USA). RNA concentrations were measured using a Qubit^®^ RNA Assay Kit in a Qubit^®^ 2.0 Fluorometer (Life Technologies, CA, USA). RNA integrity was assessed using the RNA Nano 6000 Assay Kit on the Agilent Bioanalyzer 2100 system (Agilent Technologies, CA, USA). High quality RNA samples (RIN number > 7.0) were selected for preparing a cDNA library.

For library construction, NEBNext^®^ Ultra^™^ RNA Library Prep Kit for Illumina^®^ (E7530, NEB, USA) was used according to manufacturer’s recommendation. Briefly, mRNA was purified using poly-T oligo-attached magnetic beads and fragmented into small fragments. Random hexamer primers and M-MuLV Reverse Transcriptase (RNase H) were used for first-strand cDNA synthesis. Subsequently, DNA polymerase I and RNase H were used to synthesize second-strand cDNAs. After end-repair and adapter-ligation, the products were amplified using PCR with Phusion High-Fidelity DNA polymerase and purified using the AMPure XP system (Beckman Coulter, Beverly, USA). Library quality was assessed on the Agilent Bioanalyzer 2100 system. A cBot Cluster Generation System using a TruSeq PE Cluster Kit v3-cBot-HS (Illumina, San Diego, CA, USA) was used to generate clusters of index-codes according to the manufacturer’s instructions. The cDNA libraries were sequenced on an Illumina HiSeq X Ten platform (Illumina), and paired-end reads were generated.

### Quality control, *de novo* transcriptome assembly and gene functional annotation

Raw data (reads) were obtained using the Illumina HiSeq X Ten platform. After removing adapters, ploy-Ns and low quality reads, clean reads were obtained. At the same time, Q20, Q30, GC-content and the sequence duplication level of the clean data were calculated. Trinity [27] was used to assemble the transcriptome. To obtain comprehensive information, gene functional annotations of seven databases, NCBI non-redundant protein sequence (Nr), NCBI non-redundant nucleotide sequence (Nt), Protein family (Pfam), euKaryotic Ortholog Groups (KOG), Swiss-Prot (a manually annotated and reviewed protein sequence database), KEGG Ortholog (KO) and gene ontology (GO), were carried out.

### Differential expression analysis and functional enrichment

The DESeq R package (1.10.1) was used to perform a differential gene expression analysis between mock and Se-treatment groups [28]. DESeq software provides a statistical program for identifying differentially expressed genes (DEGs) in gene expression data based on a negative binomial distribution model. The resulting P values were adjusted using the Benjamini and Hochberg’s approach for controlling the false discovery rate. Genes with an adjusted P-value < 0.05 and |log2FoldChange |> 1 as assessed by the DESeq analysis were assigned as differentially expressed. A GO enrichment analysis of the DEGs was implemented in the GOseq R package based on the Wallenius non-central hyper-geometric distribution, which can adjust for gene length bias in DEGs [29]. Enriched KEGG pathways were determined using KOBAS software [30].

### Metabolite extraction for the untargeted metabolomics analysis

The stem samples from the mock or Se-treatment of celery seedlings (100 mg each, n = 8) were ground in liquid nitrogen and transferred to centrifuge tubes. An aliquot of 400 μL of 80% aqueous methanol solution was added to each tube, vortexed and allowed to stand at -20°C for 60 min. Then, samples were centrifuged at 14,000 × *g* for 10 min at 4°C. A certain amount of supernatant was placed in a new centrifuge tube, vacuum freeze-dried, precipitated with 100 μL of 80% methanol for dissolution, vortexed and centrifuged at 14,000 × *g* at 4°C for 15 min. The supernatants were injected into an ultra-high performance liquid chromatography-tandem mass spectrometry (UHPLC-MS/MS) system for analysis. To control the quality of the experiment, QC samples were prepared and processed as alongside the samples. The QC samples contained an equal mixture of experimental samples used to balance the UHPLC-MS/MS system and monitor the state of the instrument. The system’s stability was evaluated throughout the experimental process.

### UHPLC-MS/MS analysis for the untargeted metabolites

The chromatographic separations of the samples were performed using a Vanquish^™^ UHPLC system (ThermoFisher Scientific, Waltham, MA, USA). An Accucore HILIC column (50 mm × 2.6 mm, 2.1 μm; ThermoFisher Scientific) was used for the reversed phase separation. The column oven was maintained at 40°C, and the flow rate was 300 μL/min. For positive ion mode, the mobile phase consisted of solvent A (0.1% formic acid + 95% acetonitrile + 10 mM ammonium acetate) and solvent B (0.1% formic acid + 50% acetonitrile + 10 mM ammonium acetate). For negative ion mode, the mobile phase consisted of solvent A (95% acetonitrile + 10 mM ammonium acetate, pH 9.0) and solvent B (50% acetonitrile + 10 mM ammonium acetate, pH 9.0). Gradient elution conditions were set as follows: 0–1 min, 98% A; 1–17 min, 2% to 50% B; 17–17.5 min, 50% A; 17.5–18 min, 50% to 98% A; 18–20 min, 98% A.

A high-resolution tandem mass spectrometer QE HF-X (ThermoFisher Scientific) was used to detect metabolites eluted from the column. The scan range was from 100 to 1,500 *m/z*. The electrospray ionization source was set as follows: spray voltage, 3.2 kV; sheath gas flow rate, 35 Arb; aux gas flow rate, 10 Arb; capillary temperature, 320°C and polarity, positive and negative.

### Identification of metabolites

The obtained MS data files were imported into the Compound discoverer database search software, and parameters, such as retention time and mass to charge ratio, were used for screening. Then, peak alignments of different samples were conducted based on 0.2 min retention time deviations and 5 ppm quality deviations to increase the accuracy of the identifications. In accordance with a quality deviation of 5 ppm, signal strength of 30%, signal-to-noise ratio of 3, a 100,000 minimum signal strength and ion peaks, as well as extracted information, such as peak areas for quantitative assessments and integrated target ions, a prediction formula was attained, and the results were compared with the mzCloud database (https://www.mzcloud.org/). The quantitative results were normalized using the QC sample, and the final data identification and quantitative assessments were obtained.

## Results

### Illumina sequencing and de novo assembly

Six samples from mock and Se-treated celery stems were collected for transcriptome sequencing. A total of 307,027,326 raw reads were generated. Approximately 297,911,046 (97.03%) clean reads (44.68 Gb) were obtained after filtering adapters and low-quality reads (Table 1). For mock samples, 54,448,562 (97.72% of the raw reads), 53,072,190 (96.75% of the raw reads) and 53,286,580 (97.70% of the raw reads) clean reads were obtained from the three replicates, respectively. For Se-treated samples, 46,365,002 (96.30%), 41,053,980 (97.41%) and 49,684,732 (97.34%) clean reads were obtained from the three replicates, respectively. Pair-wise Pearson’s correlation coefficients of six samples indicated the high repeatability of the sequencing data (S1 Fig).

**Table 1:** Sequencing output statistics of six samples.

Sample	Raw Reads	Clean Reads	Clean Bases	Error(%)	Q20(%)	Q30(%)	GC Content(%)
Mock1	56295942	54448562	8.17G	0.03	97.19	92.21	42.69
Mock2	54857250	53072190	7.96G	0.03	97.39	92.59	42.93
Mock3	54541362	53286580	7.99G	0.03	97.27	92.33	42.48
Se1	48148620	46365002	6.95G	0.03	97.27	92.33	42.5
Se2	42143422	41053980	6.16G	0.03	97.12	92.02	42.81
Se3	51040730	49684732	7.45G	0.03	96.93	91.61	42.57

**Fig 1:**
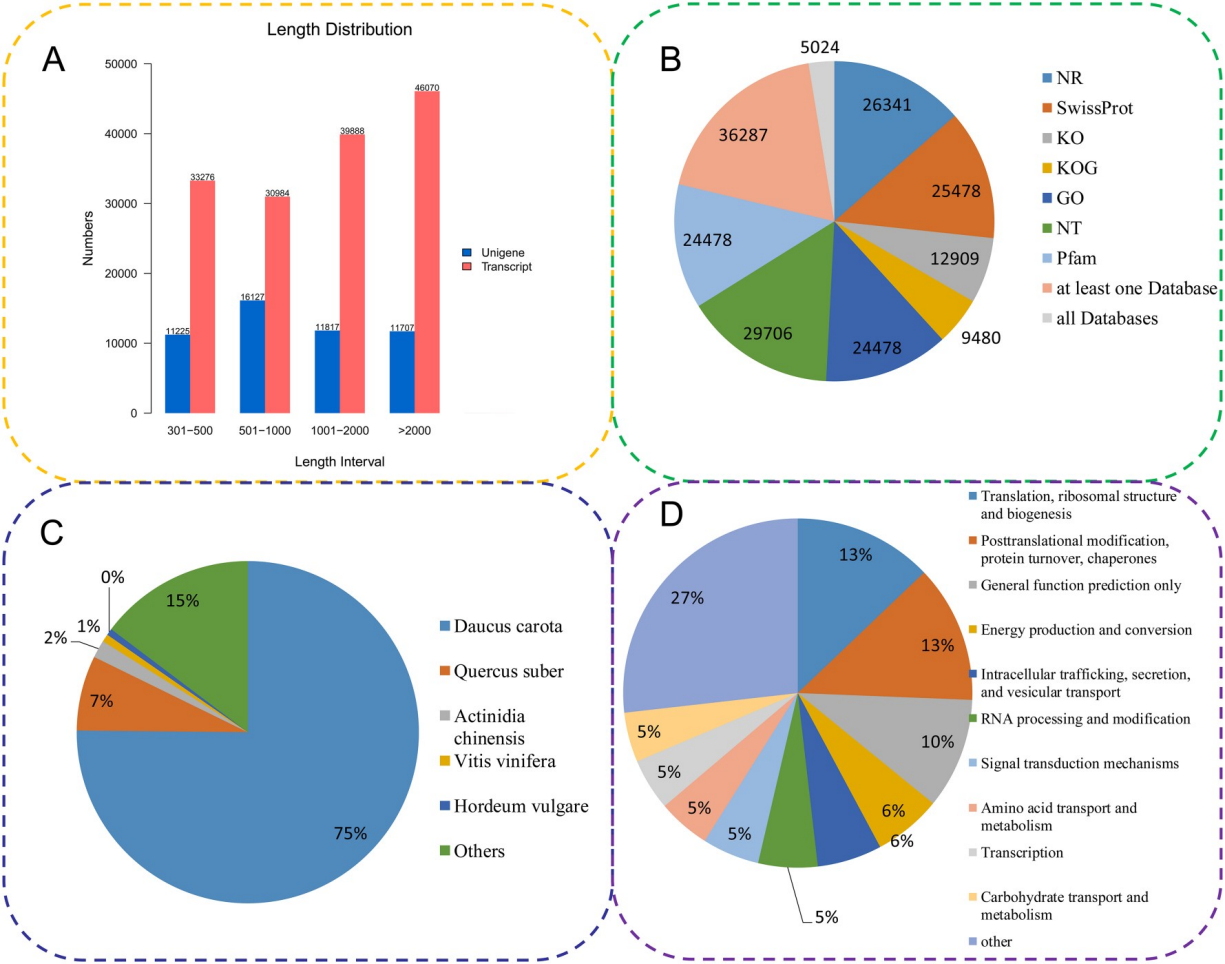
Characteristics of celery unigenes generated by Illumina sequencing. (A) The length distribution of all assembled transcripts and unigenes. (B) The number of unigenes annotated by seven different databases, including Nr, Nt, Swiss-Prot, KO, KOG, GO and Pfam. (C) Species distributions of the top BLAST hits for all homologous sequences. (D) KOG functional classifications of all the unigenes. Nr: NCBI non-redundant protein sequences; Nt: NCBI nucleotide sequences; KO: Kyoto encyclopedia of genes and genomes ortholog; KOG: eukaryotic ortholog groups; GO: gene ontology; Pfam: protein family.

Trinity software was used to assemble the clean reads. The longest cluster sequence was obtained using Corset hierarchical clustering for subsequent analyses. The lengths of transcripts and clustering sequences were statistically analyzed (Fig 1A). A total of 150,218 transcripts (50,876 genes) were obtained (N50 2,443 bp), with an average length of 1,629 bp. Among them, 33,276 transcripts were distributed between 200–500 bp, 30,984 transcripts were distributed between 500–1,000 bp, 39,888 transcripts were distributed between 1–2 Kb and 46,070 transcripts were greater than > 2 Kb.

### Gene functional annotation and classification

To determine the functions of assembled unigenes in celery, all the unigene sequences were queried against seven protein databases. In total, 26,341 and 29,706 unigenes were annotated in the Nr and Nt databases, respectively. In total, 12,909 unigenes displayed significant similarities to known proteins in the KO database. Additionally, 25,478 unigenes were annotated in the Swiss-Prot database, and 24,478 and 24,478 unigenes were annotated in the Pfam and GO databases, respectively (Fig 1B). The gene functional information of celery and related species was obtained using comparisons with the Nr database. The unigene sequences of celery were most similar to the gene sequences from *Daucus carota*, *Quercus suber*, *Actinidia chinensis*, *Vitis vinifera* and *Hordeum vulgare* (Fig 1C). A total of 9,480 unigenes were annotated using the KOG database, and they clustered into 25 functional categories. The largest category was “Translation, ribosomal structure and biogenesis” (1,352 unigenes, 27%) (Fig 1D).

**Fig 2:**
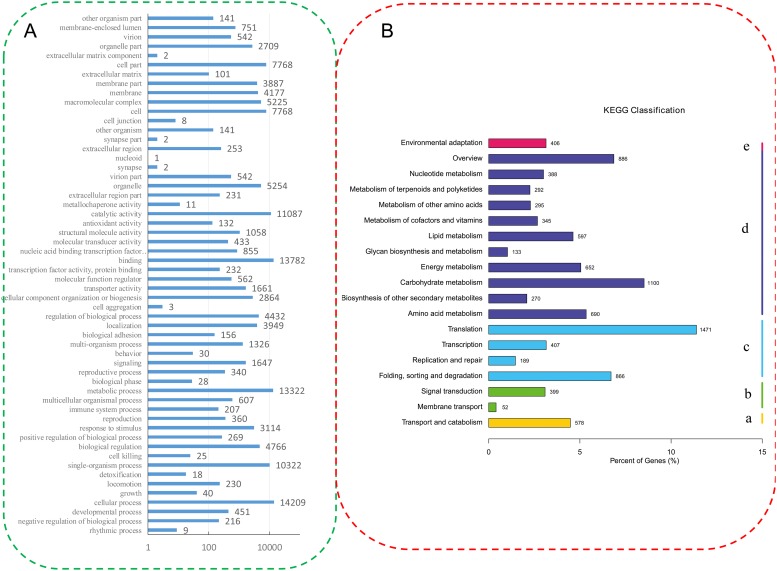
GO (A) and KEGG (B) functional classifications of the annotated unigenes in celery. The unigenes were distributed into three GO categories: biological process, cellular component and molecular function. The unigenes were divided into five KEGG groups: metabolism (a), genetic information processing (b), environmental information processing (c), cellular processes (d) and organismal systems (e).

Based on the GO annotations, 24,478 unigenes were classified into 67 functional subgroups, including 26 in “biological process,” 10 in “molecular function” and 22 in “cellular component” (Fig 2A). In the biological process category, “cellular process” was the most highly represented. In the molecular function category, “binding” was the largest subgroup, and in the cellular component category, “cell” and “cell part” contained the greatest numbers of unigenes. A total of 12,353 unigenes were grouped into 5 KEGG Pathway Hierarchy1 and 19 main Pathway Hierarchy2 (Fig 2B). The “Metabolism” categories contained the greatest numbers of unigenes, with “Carbohydrate metabolism” being the most highly represented.

### Differential gene expression and functional analyses

**Fig 3:**
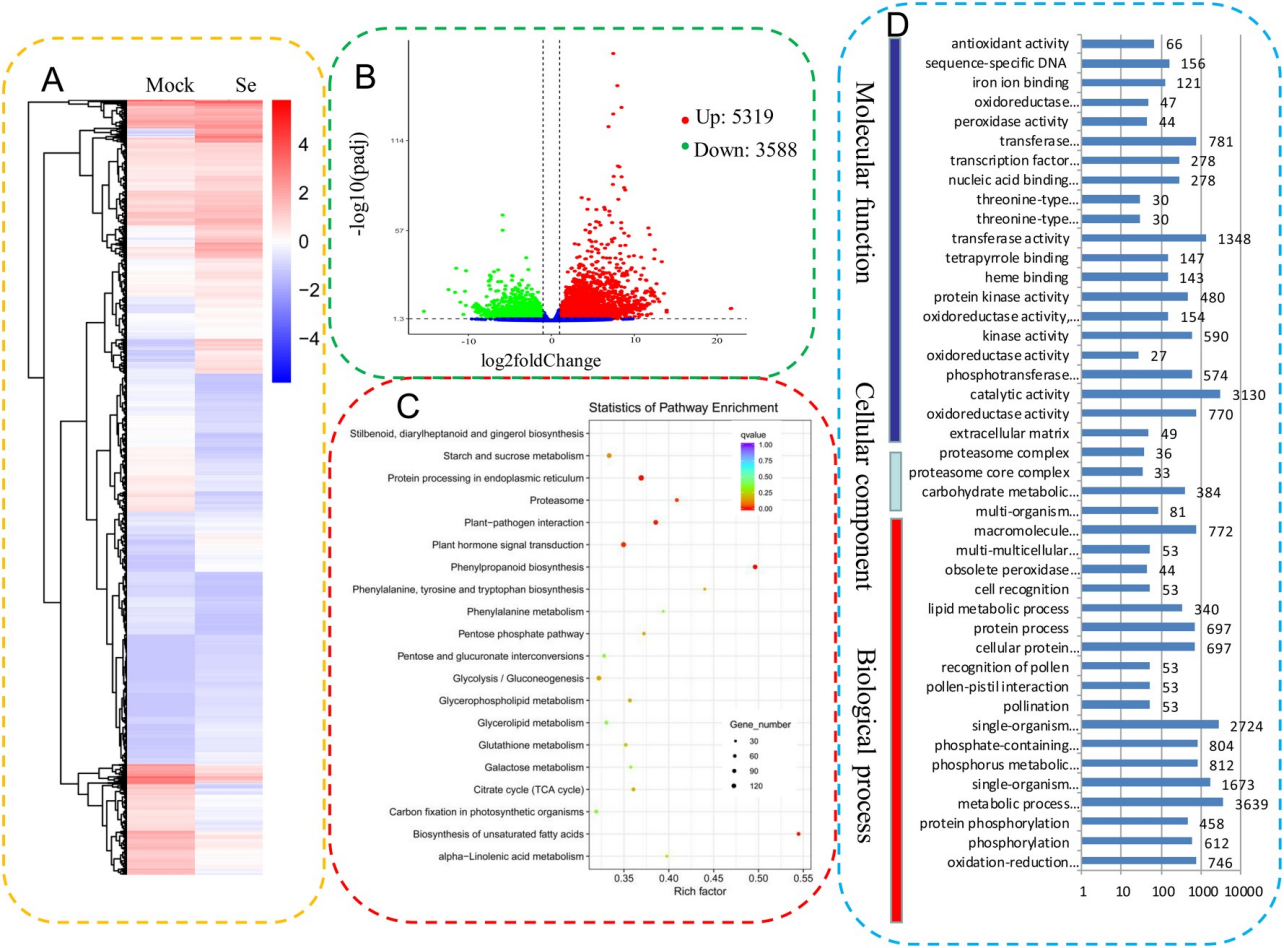
Identification of the DEGs between the mock and Se-treated celery samples. (A). Cluster diagram of differential gene expression. The color ranges from red to blue, corresponding to large to small log10 (fragments per kilobase of exon per million reads mapped +1) values. (B). Significance analysis of the DEGs as assessed by volcano plots. (C). KEGG enrichment analysis of the DEGs. The top 20 statistics of KEGG pathway enrichment are shown. The size of the q value is represented by the dot color. The smaller the q value, the closer the color is to red. The number of different genes contained in each pathway is represented by the dot size. (D). GO enrichment analysis of the DEGs.

Transcriptional responses to the Se treatments were acquired by comparing the transcriptomes of the mock and treated celery. Fragments per kilobase of exon per million reads mapped was used to estimate the gene expression level. Differential gene expression profiles after Se treatments are shown as heatmaps (Fig 3A and S1 Table). A total of 8,907 DEGs, including 5,319 up- and 3,588 downregulated genes, were identified (Fig 3B). A detailed KEGG enrichment analysis of the DEGs is shown in Fig 3C and S2 Table. “Phenylpropanoid biosynthesis” was the most enriched pathway. “Protein processing in endoplasmic reticulum” contained the greatest number of unigenes (123 unigenes), followed by “Plant hormone signal transduction” (109 unigenes). A total of 24 sulfur and selenocompound metabolic genes were differentially expressed (Table 2). The GO enrichment of DEGs is shown as a histogram (Fig 3D). In the molecular function category, “transferase activity” was the most highly represented (1,348 DEGs). In the cellular component category, “extracellular matrix” contained the greatest number of unigenes (49 DEGs). In the biological process category, the “metabolic process” was the most highly represented (3,639 DEGs).

**Table 2:** The genes related with sulfur and selenocompound metabolism in the stems of celery plants.

GeneID	KO ID	Gene Description	KO Name	log2FC	q value
Cluster-1975.12663	K00384	thioredoxin reductase	trxB, TRR	1.3422	0.0001607
Cluster-26621.0	K00384	thioredoxin reductase 1	trxB, TRR	2.8927	6.3755E-12
Cluster-1975.6602	K00392	Sulfite reductase 1like	sir	2.7681	0.0031039
Cluster-1975.10040	K00549	5-methyltetrahydropteroyltriglutamate-homocysteine methyltransferase	metE	1.7232	0.017959
Cluster-1975.10039	K00549	5-methyltetrahydropteroyltriglutamate-homocysteine methyltransferase-like	metE	2.4525	0.0015574
Cluster-19378.0	K00640	serine acetyltransferase 1	cysE	9.9727	1.5467E-11
Cluster-1975.622	K00640	serine acetyltransferase 1	cysE	6.6463	4.0559E-17
Cluster-9630.0	K01082	PAP-specific phosphatase, mitochondrial	cysQ, MET22, BPNT1	-1.7109	0.000038524
Cluster-253.0	K01739	putative trans-sulfuration enzyme	metB	6.1894	0.027758
Cluster-1975.708	K01761	methionine gamma-lyase	E4.4.1.11	2.2997	0.049269
Cluster-1975.14310	K01874	hypothetical protein DCAR_026633	MARS, metG	1.1911	0.0019799
Cluster-1975.2297	K05907	5'-adenylylsulfate reductase 3	APR	6.3746	3.2312E-11
Cluster-1975.2154	K05907	5'-adenylylsulfate reductase 3	APR	7.5437	1.4671E-12
Cluster-1975.39	K08247	Aminotransferase	E2.1.1.12	1.9072	0.000076199
Cluster-23972.0	K08247	hypothetical protein DCAR_001729	E2.1.1.12	-3.7045	2.1307E-06
Cluster-24206.0	K08738	Cytochrome c	CYC	5.2202	0.00087537
Cluster-1975.10037	K13034	hypothetical protein DCAR_005355	ATCYSC1	2.8054	1.4219E-06
Cluster-1975.5913	K13811	ATP-sulfurylase 3	PAPSS	2.1472	0.0064403
Cluster-1975.5914	K13811	ATP-sulfurylase 3	PAPSS	1.5553	0.0096365
Cluster-1975.2031	K13811	hypothetical protein DCAR_027249	PAPSS	5.254	5.9058E-15
Cluster-25056.1	K13811	ATP sulfurylase 2	PAPSS	1.388	0.0054543
Cluster-10657.0	K17069	homocysteine synthase-like	MET17	5.3604	0.0031751

### Untargeted metabolite profiling analysis

**Fig 4:**
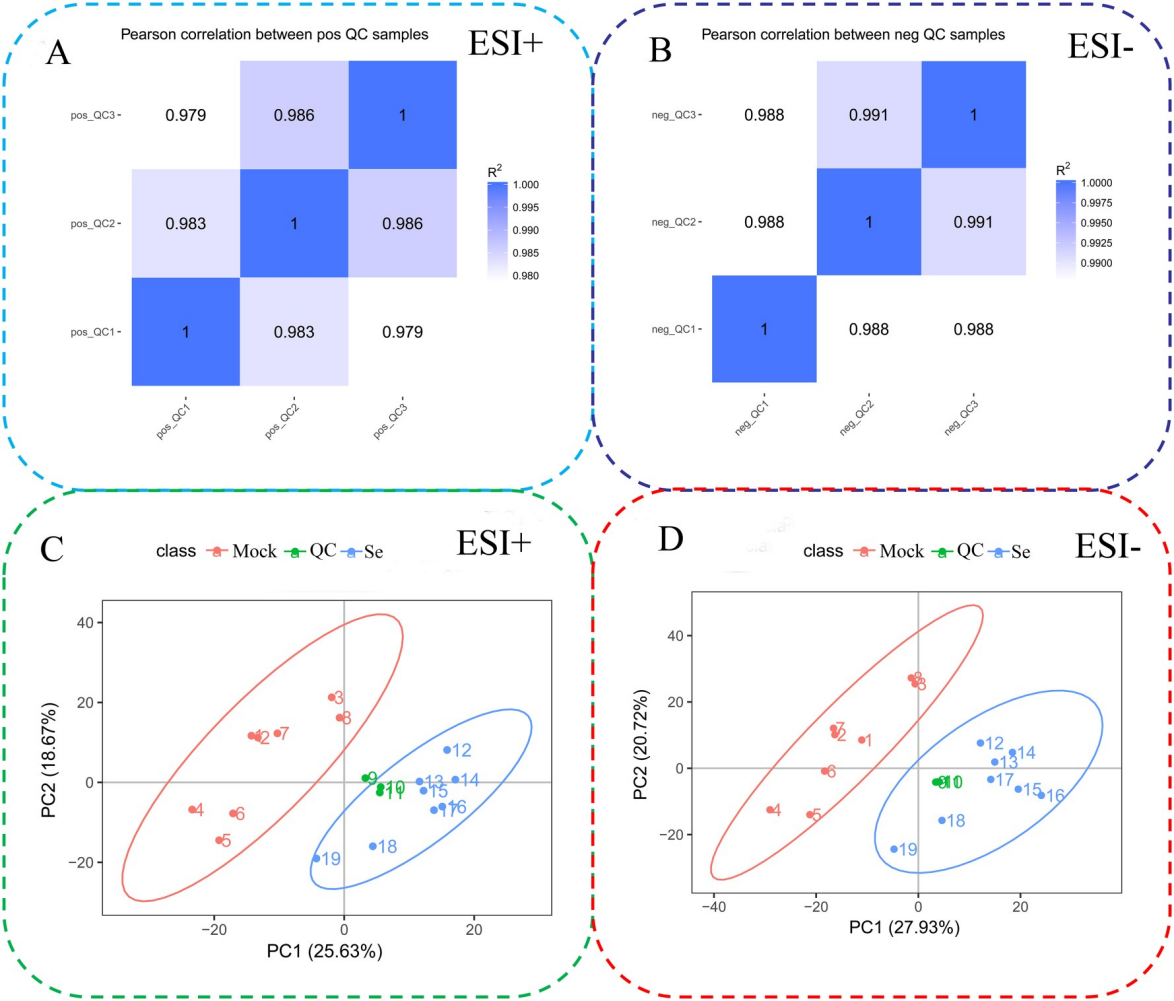
QC sample correlations and principal component analyses (PCAs) of 19 celery samples. QC sample correlation in electrospray ionization positive (ESI+) (A) and negative (ESI−) modes (B). QC samples were used to determine the instrumental state before injection and to evaluate the stability of the system during the whole experiment. PCAs of the ESI+ (C) and ESI−(D) data.

To study the changes of metabolites after the Se treatment, an untargeted metabolomic approach was used (n = 8). Compound discoverer software was used to search and analyze the data. Using the mzCloud database, the compounds were identified and confirmed by spectrogram comparison. A total of 1,774 metabolites were identified (Table 3 and S3 Table). The QC samples’ correlations increased in tandem with the whole method’s stability and data quality (Fig 4A). A principal component analysis was carried out to assess the total metabolic differences between samples in each group and the degree of variation among samples in the group (Fig 4B). We also measured some physiological parameters between treated and non-treated plants. The results showed that the contents of hydrogen peroxide and malondialdehyde increased, while the contents of chlorophyll and total flavonoids decreased under selenium treatment (S2 Fig).

**Table 3:** Identified metabolite statistics using UPLC-MS/MS analysis.

Compared Samples	Num. of Total Identified.	Num. of Total significant difference	Num. of significant Up	Num. of significant down
Se.vs.Mock_pos	858	99	87	12
Se.vs.Mock_neg	916	138	126	12

**Fig 5:**
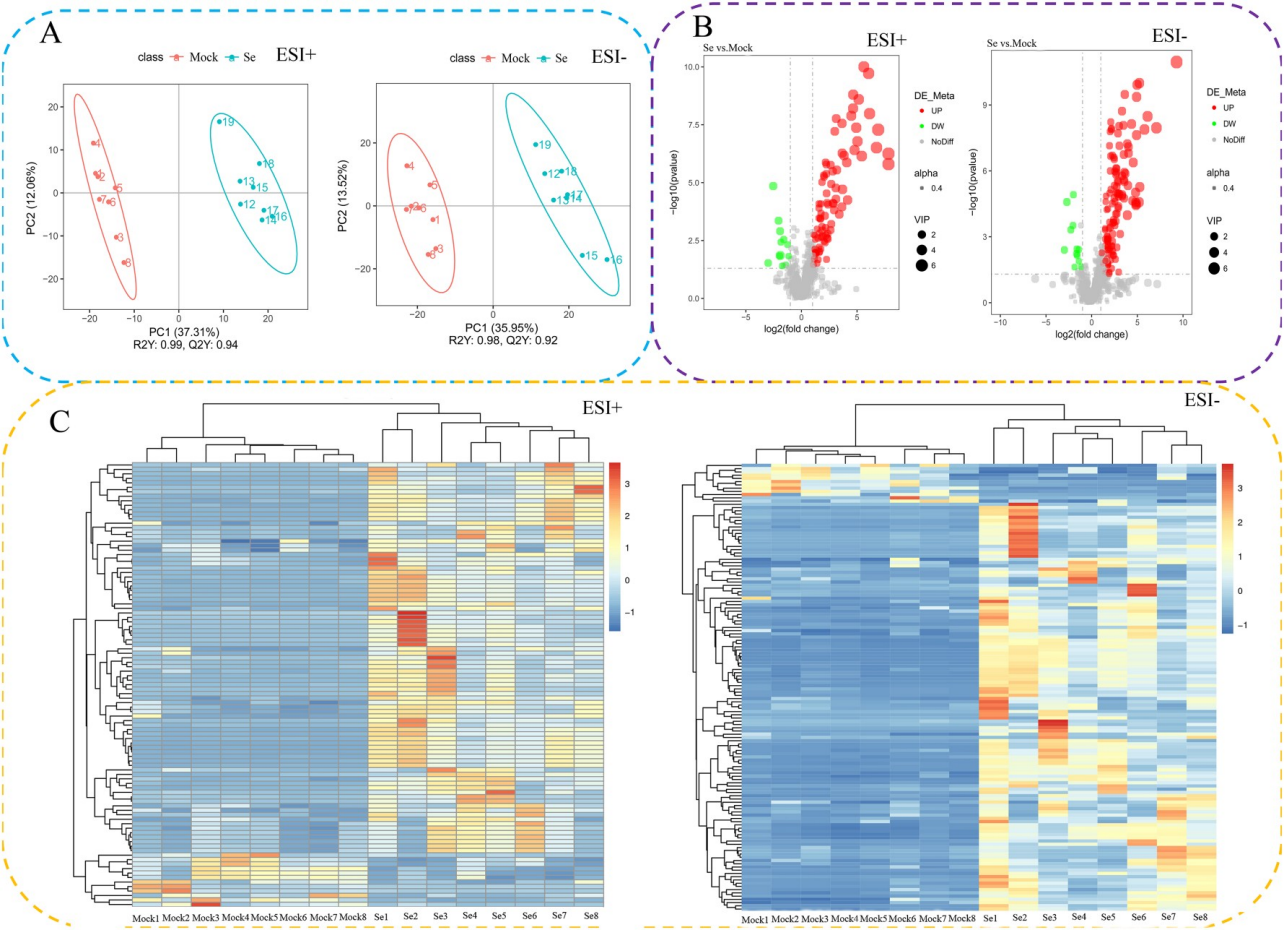
Differential metabolites analysis. (A) The PLS-DA score plot shows groupings of Se-treated vs. mock samples in both ESI+ and ESI− modes. The abscissa represents the score of the sample for the first principal component; the ordinate represents the score of the sample for the second principal component; R2Y is the interpretation rate of the second principal component of the model; Q2Y is the prediction rate of the model. (B) Volcanic map of differential metabolites in both ESI+ and ESI− modes. Black represents metabolites having no significant differences, red represents upregulated metabolites, green represents downregulated metabolites, and the dot size represents the Variable Importance in the Projection Value. (C). Heat map and cluster analyses of mock and Se-treated celery varieties at the metabolome level in both ESI+ and ESI− modes. Hierarchical clustering was used to analyze the differentiated metabolites. The relative quantitative values of differentiated metabolites were transformed into Z values and clustered. Different color regions represent different clustering information. The metabolic expression patterns within the same group are similar, which indicates that they may have similar functions or participate in the same biological process.

A partial least squares discrimination analysis uses a partial least squares regression to establish a model of the relationships between metabolite expression and sample categories. The results showed a distinct separation between mock and Se-treated sample groups (Fig 5A). These generated models explained variation values from the seven-fold cross-validated R2Y≥0.98 and Q2Y≥0.92. The statistical analysis identified 237 significantly differentially accumulated metabolites (SDAMs), including 213 up- and 24 down-regulated SDAMs in both positive and negative electrospray ionization modes based on the following criteria: Variable Importance in the Projection > 1.0, 0.5 < fold change > 2.0 with P value < 0.05 (Fig 5B and S4 Table). An overview of the SDAM profiles of mock and Se-treated celery is shown in Fig 5C.

### Integrated analyses of transcriptomic and metabolomic changes involved in vital biological pathways

**Fig 6:**
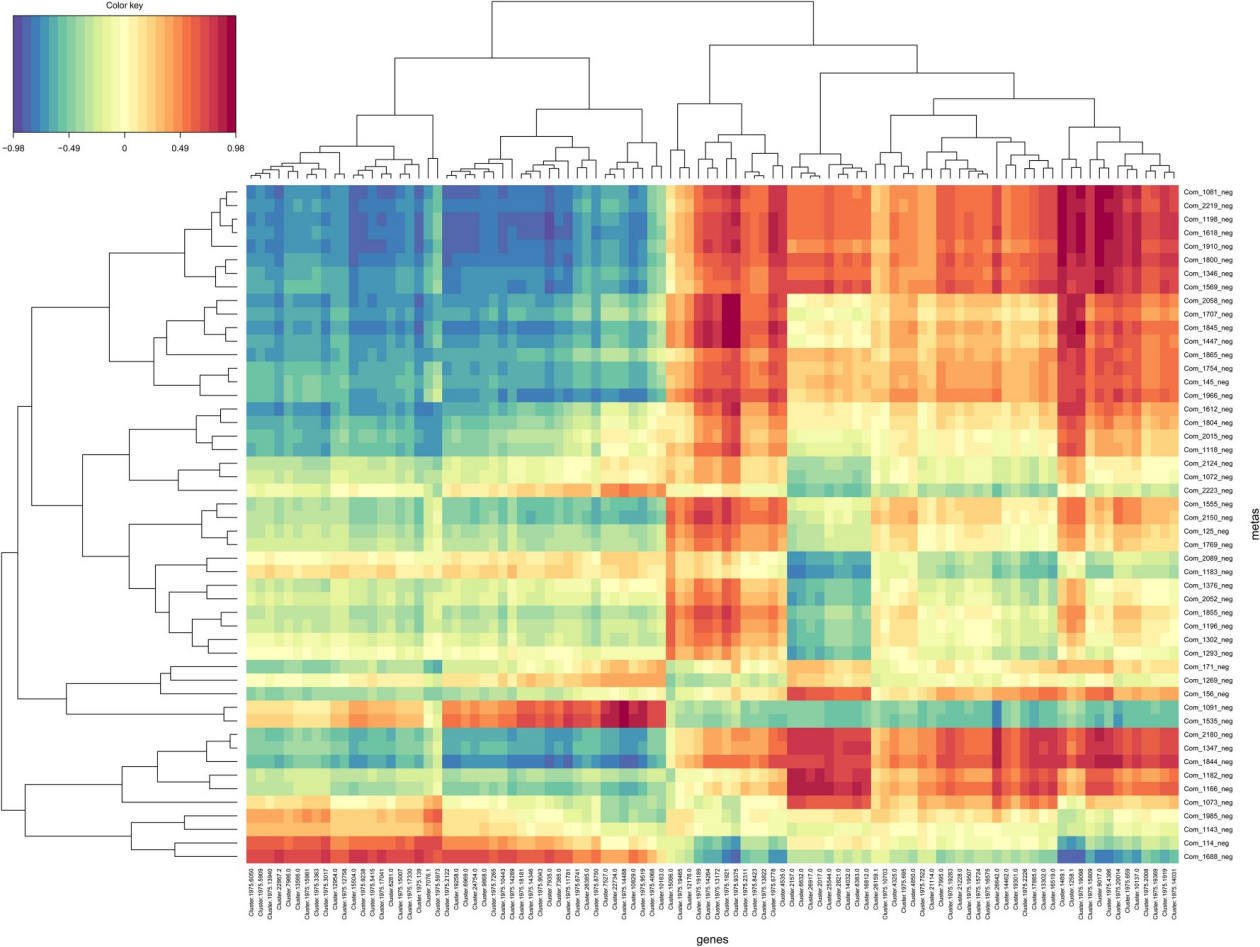
Results of the correlation analysis between DEGs and DAMs. There were 50 of the former and 100 of the latter. Red represents a positive correlation and blue represents a negative correlation.

**Fig 7:**
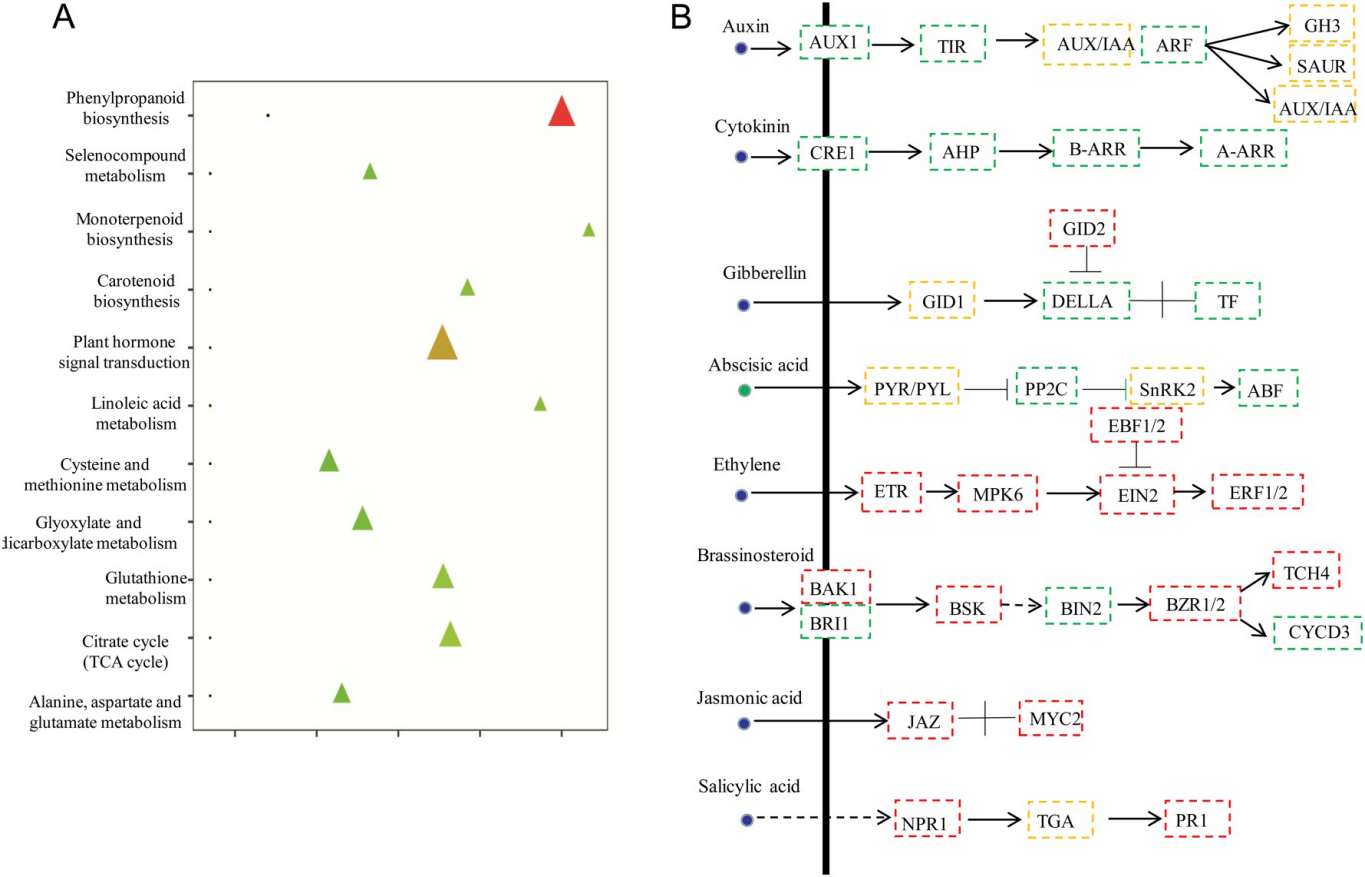
Association analysis of metabolomic and transcriptomic pathways. (A). Analysis of KEGG pathways in the metabolome and transcriptome. Dots represent metabolic data, and delta signs represent transcribed data. (B). Identification and differential analysis of the hormonal network between mock and Se-treated celery. Red boxes indicate the predominantly expressed genes in Se-treated celery. Green boxes indicate the down-regulated genes in Se-treated celery. Yellow boxes represent families that contained up- and downregulated members. Green circles represent decreasing metabolites.

To obtain more information regarding the physiological changes of celery under Se stress, we analyzed the relationships between differential gene expressions and changes in Se-responsive metabolites. The results of the correlation analysis between gene expression levels and metabolites are shown in Fig 6 and S5 Table. To understand the changes in the metabolic regulatory network of celery in response to Se stress, we conducted a correlation analysis of enriched KEGG pathways of DEGs and SDAMs (S6 Table). As shown in Fig 7A, there are 11 pathways shared by the metabolome and the transcriptome. The “Plant hormone signal transduction” pathway contained the greatest number of DEGs (109 unigenes). Many DEGs participate in various hormone-signaling pathways as indicated by the KEGG analysis. The summary of the various hormone-signaling networks in celery is shown in Fig 7B. For auxin-related DEGs, AUX1 (K13946), TIR1 (K14485) and ARF (K14486) were downregulated by the Se treatment. DEGs associated with cytokinin were also down-regulated. In the ethylene and jasmonic acid pathways, all the DEGs were upregulated. Among abscisic acid-related DEGs, PP2C (K14497) and ABF (K14432) were downregulated by Se stress. Additionally, abscisate (Com_1688_neg) was downregulated by the Se treatment.

## Discussion

Celery, an important vegetable belonging to the Apiaceae family, is widely grown in Europe and in tropical and subtropical areas [31]. The development of various omics techniques has promoted the research of celery growth and breeding. For example, a genomic database of celery, CeleryDB, was constructed for the convenience of researchers to study celery[32]. Lignin biosynthesis-related enzymes and simple sequence repeat (SSR) markers were identified using transcriptome sequencing[33,34]. A total of 71 temperature stress-responsive proteins were identified through MALDI-TOF-TOF analysis[35]. Transcriptome sequencing has become a powerful technique for elucidating new genes and their related biochemical pathways in non-model plants because of its high throughput, low cost and large data output [36]. Metabolomics is a new field in the post-genome era that reveals the changing roles of metabolites in various tissues [37]. Metabolites are the end products of cellular biological processes, and their levels can be used as indicators of plant responses to genetic and environmental changes [38]. The combination of transcriptomic and metabolic data is useful for identifying genes that may be involved in metabolic networks [39]. Se-induced genes were identified in several species using RNA-seq [40,41], but the genes related to Se uptake, accumulation and tolerance in celery remain unclear. In the present study, we combined transcriptome and metabolomics analyses to determine the differences in gene transcriptional levels and metabolic pathways between Se-treated and untreated celery. In our study, 50,876 unigenes were generated by Illumina sequencing, of which 36,287 unigenes were annotated by seven databases. Furthermore, 8,907 unigenes were identified as DEGs between Se-treated and untreated celery seedlings. A total of 1,774 metabolites were successfully obtained by UHPLC-MS/MS, of which 213 were identified as SDAMs. Our study found that many new genes and metabolites were involved in many biochemical pathways, including Se tolerance and metabolism.

Se is mainly transported as selenate and selenite through S and P transporters in plants, respectively. Interestingly, the expression levels of seven phosphate transporters were increased by selenate, including the phosphate and inorganic phosphate transporters and mitochondrial phosphate carrier genes. Additionally, the expression levels of three of four sulfate transporters were increased by selenate (Table 4). Thus, P and S transporters were probably the main pathways for the uptake and transport of selenate in celery. Se can form selenocysteine and selenomethionine through the S-assimilation pathway[42]. Therefore, we further analyzed the DEGs associated with sulfur and selenocompound metabolism. ATP sulfurylase (APS), a first and rate-limiting enzyme, mediates the binding of selenate and ATP to form on adenosine-5-phosphoselenate [36]. In the present study, the expression levels of four APS unigenes increased in celery plants. APS2, a cytoplasmic protein in *A*. *thaliana* plays a major role in the assimilation of selenate [43]. Adenosine-5-phosphoselenate is reduced to selenite by adenylylsulfate reductase.

**Table 4:** The DEGs related with phosphate and sulfate transporters.

Gene_id	Protein Description	log2FC	q value
Cluster-23123.0	phosphate transporter PHO1 homolog 3-like isoform X1	-1.5412	0.0059557
Cluster-437.1	phosphate transporter PHO1 homolog 3-like	6.3006	1.17E-23
Cluster-1975.16237	inorganic phosphate transporter 1-4-like isoform X1	3.3445	4.28E-08
Cluster-25918.2	phosphate transporter PHO1 homolog 3-like	3.5771	0.013098
Cluster-350.0	phosphate transporter PHO1 homolog 3-like	5.8071	0.0041555
Cluster-1975.14365	mitochondrial phosphate carrier protein 3, mitochondrial-like	3.8973	9.79E-10
Cluster-17574.0	mitochondrial phosphate carrier protein 2-like	5.1274	0.0023377
Cluster-1975.17837	sulfate transporter 1.3-like	10.262	3.63E-21
Cluster-1975.5238	sulfate transporter 3.4	-2.1611	7.23E-06
Cluster-1833.0	sulfate transporter 3.5	7.1798	0.00049882
Cluster-1975.2010	sulfate transporter 4.1, chloroplastic-like	1.5402	0.035202

Plant hormones are particularly important in responses to stresses and in ionic homeostasis [44–46]. In this study, a large number of DEGs were identified in relation to plant hormones. Auxin and its related genes are involved in the homeostasis of various ions [47,48]. For example, OsABCB14, a rice auxin influx transporter, participates in Fe homeostasis [48]. In *Astragalus chrysochlorus* (a Se accumulating plant), IAA30-like, ARF2-like and ARF5-like are upregulated after selenate treatments [41]. Contrary to these results, AUX1 (K13946), TIR1 (K14485) and ARF (K14486) were downregulated by the Se treatment. This is probably because different plants absorb different amounts of Se and have different metabolic mechanisms. For example, in *Arabidopsis*, selenate treatments reduce the auxin-reactive protein level and thus reduce plant development [49], which is consistent with our results. Our physiological data showed that the contents of H_2_O_2_ and MDA increase under Se treatment. The increased production of reactive oxygen species results in abscisic acid to playing a more important role in abiotic stress responses [44]. In addition, these hormones can affect the absorption and assimilation of S [50], which may help plants prevent Se from replacing S in proteins [42].

Based on highly accurate modern MS, the metabonomics analysis method can directly analyze small molecule metabolites qualitatively and quantitatively, and the activities of gene expression products can be understood at the metabolic level [51]. Lobinski’s team has conducted in-depth studies on a Se-rich yeast metabolome and established a set of relatively complete analysis methods for the Se metabolome [51–54]. A comprehensive metabolite analysis is important for describing the final nutritional value of a celery stem. The untargeted metabonomics approach provides the opportunity to systematically analyze the metabolic changes in celery under different treatment conditions. In our study, 1,774 annotated metabolites, which belonged various metabolic pathways, were identified. In total, 10 SDAMs were classified into four significantly enriched KEGG pathways (P < 0.05), “Metabolism of xenobiotics by cytochrome P450,” “Steroid hormone biosynthesis,” “Phenylpropanoid biosynthesis” and “Biosynthesis of phenylpropanoid.” Phenolic compounds, such as phenylpropanoid and flavonoids, are the most prominently produced secondary plant metabolites, and they have antioxidative activities and protect cells from biological and abiotic stresses, including infection, injury, ultraviolet radiation, ozone, pollutants and herbivores [55–57]. Total flavonoids was reduced by Se theatment. The changes in these metabolite levels may result from increased oxidative stress caused by Se stress.

## Conclusion

In conclusion, we used transcriptome and non-targeted metabolomic techniques to study differences in gene expression and metabolite accumulation-related responses to Se exposure. In total 8,907 DEGs, including 5,319 up- and 3,588 downregulated genes, were identified. Additionally, 11 KEGG pathways changed both at transcriptome and metabolic levels under Se-stress conditions. Plant hormones and phenylpropanoids might play important roles in plants responses to Se stress.

## Supporting information

S1 Fig Correlation coefficient and gene expression among samples. (A) Pearson correlation between samples. (B) Comparison map of gene expression level under different experimental conditions. The abscissa is the log10 (FPKM) value of the gene, and the ordinate is the density of the corresponding log10 (FPKM). Different colors represent different samples. The graph measures the differences among the samples from the overall distribution of the expression. (C) FPKM box chart. The abscissa is the name of the sample, and the ordinate is log10 (FPKM+1). The box chart of each region measures the difference between the samples from the point of view of the overall dispersion of the expression quantity, with five statistics (top-down maximum, upper quartile, median, lower quartile and minimum, respectively. (D) FPKM interval distribution.
(TIF)

S2 Fig Effects of exogenous Se treatment on hydrogen peroxide(A), chlorophyll(B), malondialdehyde(C) and flavonoids(D) in celery stems. *Indicates a signifcant difference at P < 0.05, and **Indicates a signifcant difference at P < 0.01 (Student’s t test).
(TIF)

S1 Table Detailed information of identified differentially expressed genes. (XLS)

S2 Table KEGG enrichment analysis of the DEGs. (XLS)

S3 Table Detail information of all identified metabolites. (XLS)

S4 Table Detail information of all identified SDAMs. (XLS)

S5 Table Correlation analysis between gene expression and metabolites. (XLS)

S6 Table Correlation analysis of enriched KEGG pathways of DEGs and SDAMs. (XLS)
