# Supplementary figures and images for: Comparative de novo transcriptomics and untargeted metabolomic analyses elucidate complicated mechanisms regulating celery (Apium graveolens L.) responses to selenium stimuli
Source: PLoS One. 2019 Dec 30;14(12):e0226752. doi: 10.1371/journal.pone.0226752 (PMC6936847; doi:10.1371/journal.pone.0226752)

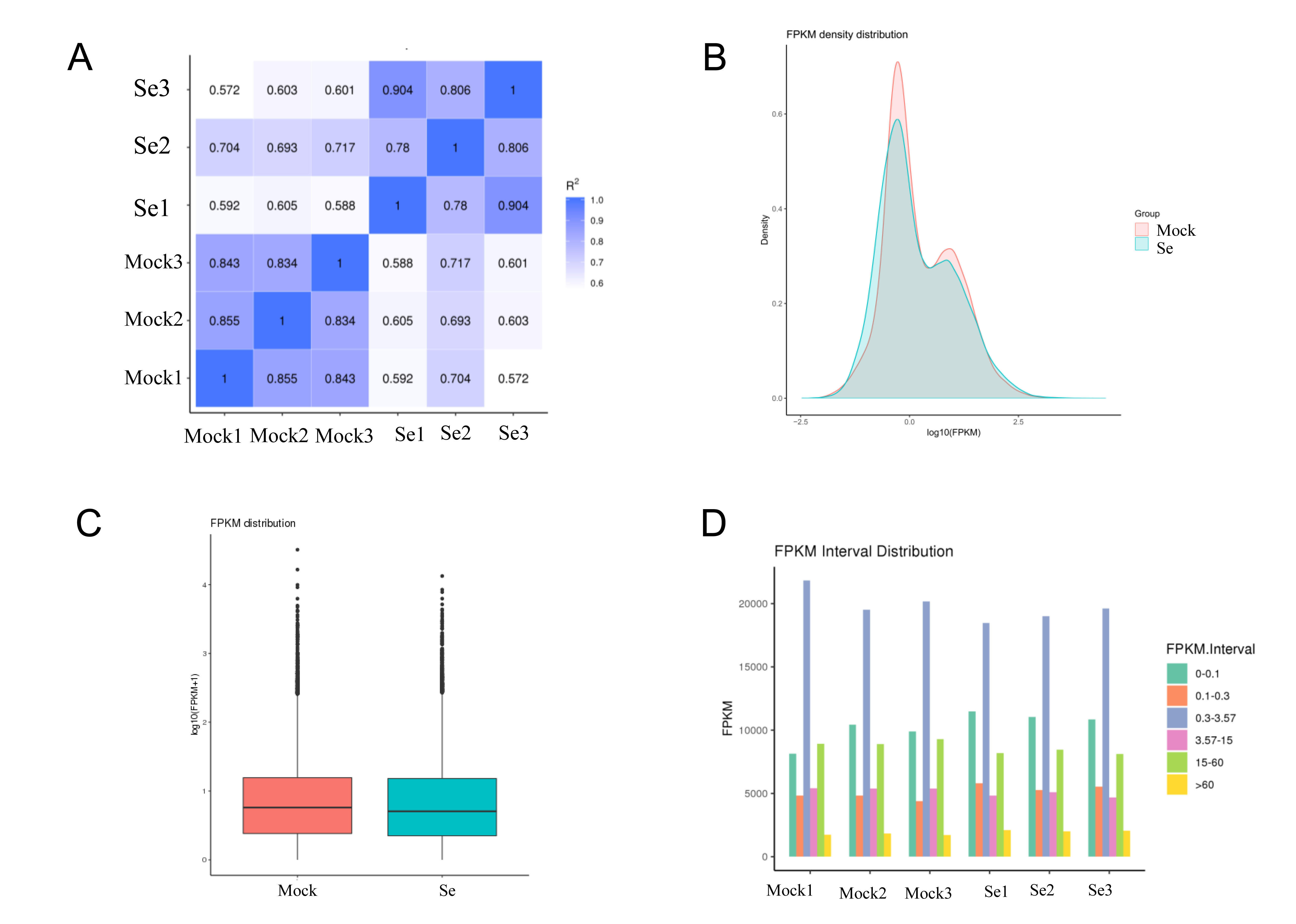

Supplement: S1 Fig — (A) Pearson correlation between samples. (B) Comparison map of gene expression level under different experimental conditions. The abscissa is the log10 (FPKM) value of the gene, and the ordinate is the density of the corresponding log10 (FPKM). Different colors represent different samples. The graph measures the differences among the samples from the overall distribution of the expression. (C) FPKM box chart. The abscissa is the name of the sample, and the ordinate is log10 (FPKM+1). The box chart of each region measures the difference between the samples from the point of view of the overall dispersion of the expression quantity, with five statistics (top-down maximum, upper quartile, median, lower quartile and minimum, respectively. (D) FPKM interval distribution. (TIF) [file pone.0226752.s001.tif]

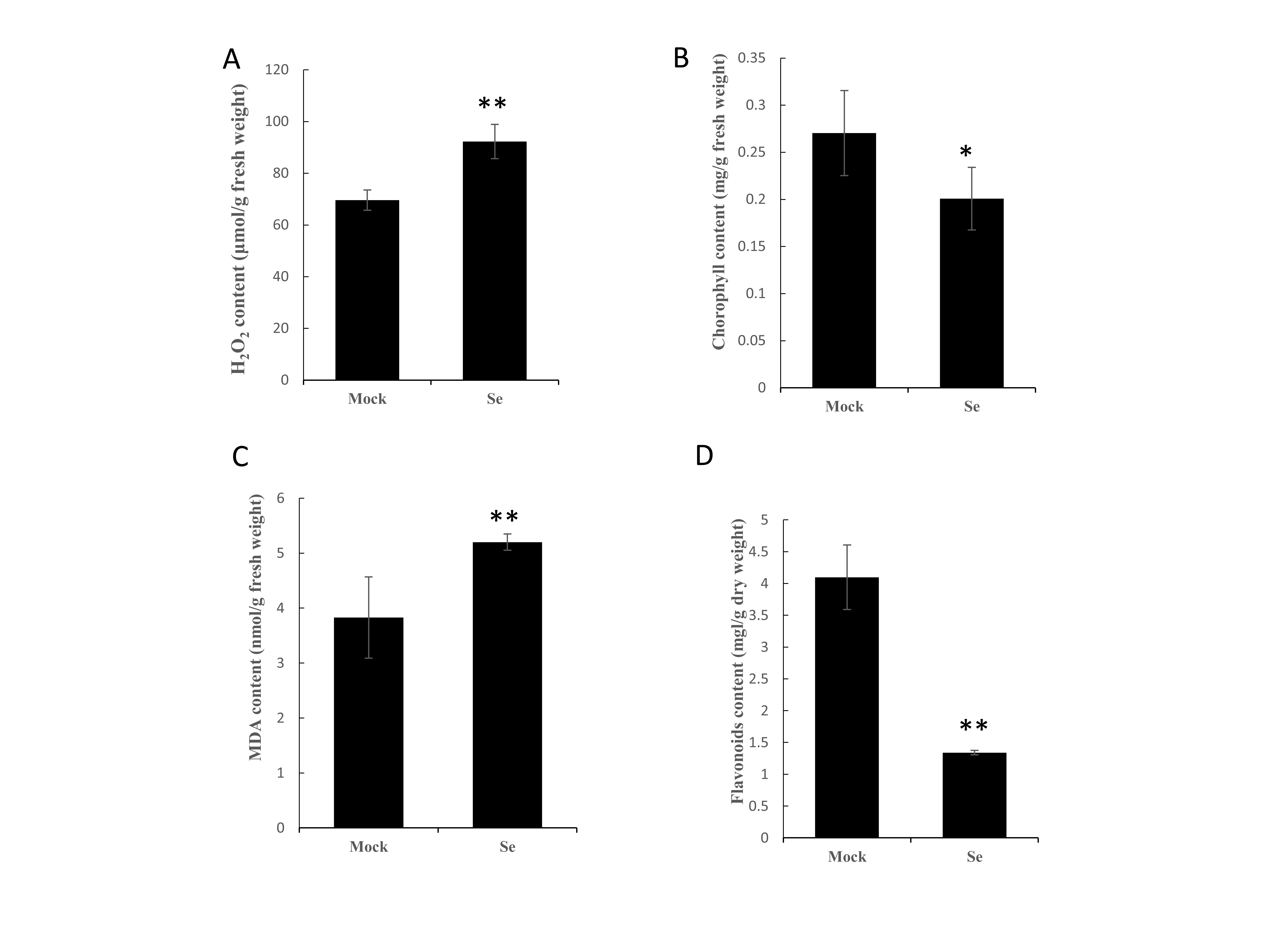

Supplement: S2 Fig — *Indicates a signifcant difference at P < 0.05, and **Indicates a signifcant difference at P < 0.01 (Student’s t test). (TIF) [file pone.0226752.s002.tif]
